# Single-incision laparoscopic cholecystectomy: How I do it?

**DOI:** 10.4103/0972-9941.72367

**Published:** 2011

**Authors:** Deepraj Bhandarkar, Gaurav Mittal, Rasik Shah, Avinash Katara, Tehemton E Udwadia

**Affiliations:** Department of Minimal Access Surgery, Hinduja Hospital, Veer Savarkar Marg, Mahim, Mumbai - 400 016, India; 1 Department of Pediatric Surgery, Hinduja Hospital, Veer Savarkar Marg, Mahim, Mumbai - 400 016, India

**Keywords:** Cholecystectomy, laparoscopy, single-port access surgery, Single-incision laparoscopic surgery

## Abstract

Single-incision laparoscopic cholecystectomy (SILC) is a relatively new technique that is being increasingly used by surgeons around the world. Unlike the multi-port cholecystectomy, a standardised technique and detailed description of the operative steps of SILC is lacking in the literature. This article provides a stepwise account of the technique of SILC aimed at surgeons wishing to learn the procedure. A brief review of the current literature on SILC follows.

## INTRODUCTION

Single-incision laparoscopic surgery (SILS) is a recent advance that has taken the surgical community by storm. Single-incision laparoscopic cholecystectomy (SILC) is perhaps the most common SILS procedure used to treat patients with gallstone disease. There are three approaches to SILC: (a) one that uses special, purpose-made access devices or ports for introducing the laparoscope and instruments which are usually, but not always, roticulating ones; (b) passing three 5-mm trocars side-by-side through the fascia after exposing a wide area via a single umbilical incision; and (c) using two trocars at the umbilicus along with suspension sutures to retract the gallbladder. Navarra *et al*. originally described a technique using transabdominal sutures to suspend the gallbladder during laparoscopic cholecystectomy (LC).[1] The technique did not gain much popularity and was not used for over 10 years. With recent interest in further minimisation of the trauma of access by reduction in the number of ports, there is a renewed interest in the use of sutures for retraction of the gallbladder – a technique known as the “puppeteer technique”. This article provides a detailed, step-by-step description of a technique of SILC using standard laparoscopic instruments.

### Indications

The indications for an SILC are the same as those for a multi-port LC (MLC), viz. biliary colic, chronic cholecystitis, and previous biliary pancreatitis or obstructive jaundice due to stones.

### Contraindications

During the learning curve of SILC grossly obese individuals, patients having undergone previous upper abdominal surgery, those with acute cholecystitis and those shown to have a small shrunken gallbladder on preoperative ultrasonography may be considered as relative contraindications. If an LC is being undertaken for a polyp, we prefer to undertake a multi-port cholecystectomy, as our technique of SILC entails placing sutures through the gallbladder and is likely to result in some spillage of bile. Further, as with a MLC, poor cardio-respiratory reserve and inability to tolerate pneumoperitoneum constitute valid contraindications. As experience is gained, SILC may be offered to the obese and those with acute cholecystitis. At all stages, it is essential to provide preoperative counselling to patients regarding the likelihood of the surgeon having to put in additional “rescue” ports or indeed converting the procedure to an open one. Such a conversion to MLC or to an open cholecystectomy is never considered a complication, but merely reflects a sound judgement on the part of the surgeon interested in safeguarding the patient’s interests.

## SURGICAL TECHNIQUE

### Instruments and equipment

**Figure 1:**
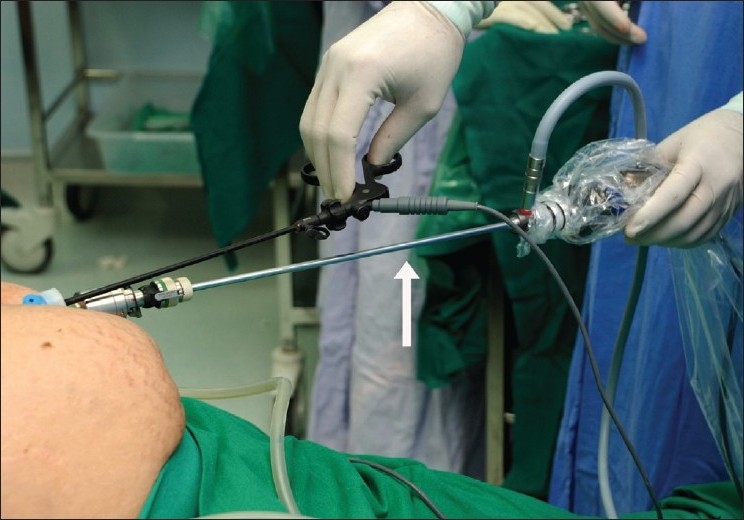
Long laparoscope (arrow) allows freedom of movement for operative instrument.

Our technique of SILC utilises conventional laparoscopic instruments and equipment. The only equipment that is different from that available in most hospitals undertaking laparoscopic surgery is a 5-mm, 30°, 51-cm long bronchoscope (Karl Storz, Tuttlingen, Germany) that we use as the laparoscope. Use of a long laparoscope moves the hand of the camera person about 8-10 inches away from the abdominal wall, thus reducing the crowding [Figure 1]. The endocamera and the light source should be of the highest quality, and we prefer the Image-1 high-definition (HD) camera along with an HD screen and a 300W Xenon light source (Karl Storz). A sharp image that allows clear distinction between tissue planes and tissue textures is essential for safe dissection. We prefer a short 5-mm, low-profile port in the ancillary position. This is either a plastic port or a short metal port designed for thoracoscopy (Karl Storz). The absence of a side gas-channel on the latter poses a handicap in that the diathermy smoke generated cannot be released easily. The smoke is vented intermittently during exchange of instruments.

### Position of the patient, team, and equipment

The patient is positioned supine on the operating table with the legs split apart and strapped firmly to the leg boards. We routinely use either anti-embolic stockings or an intermittent calf compression device. A restraining belt placed at the level of the pelvis secures the patient to the table. Both arms of the patient are placed on armboards at an angle less than 90° to the torso.

**Figure 2:**
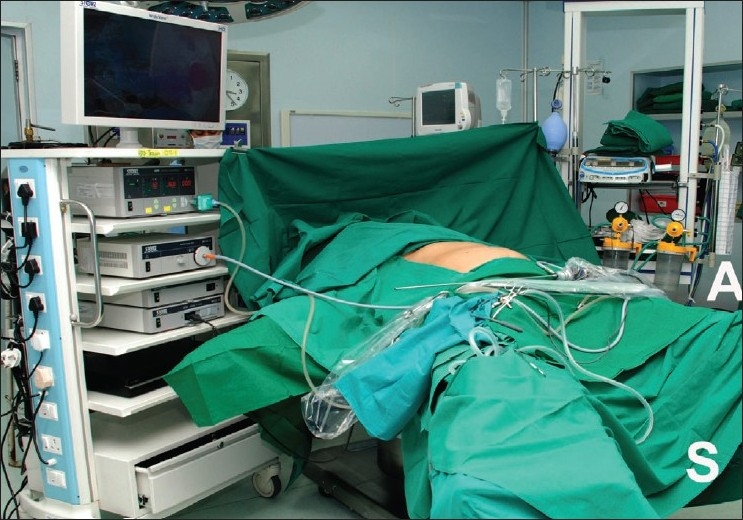
Positioning of the patient and the equipment (S = position of the surgeon, A = position of the assistant/camera person).

The surgeon stands on the left side of the patient, with the assistant opposite him during the placement of the first port. For rest of the procedure, the surgeon stands between the legs and the camera person stands to his right (near the left leg of the patient) [Figure 2]. The television trolley is placed above the patient’s right arm. The diathermy pedal is placed near the surgeon’s left foot and all tubes and cables are fixed such that they do not interfere with the camera person.

### Placement of ports

**Figure 3:**
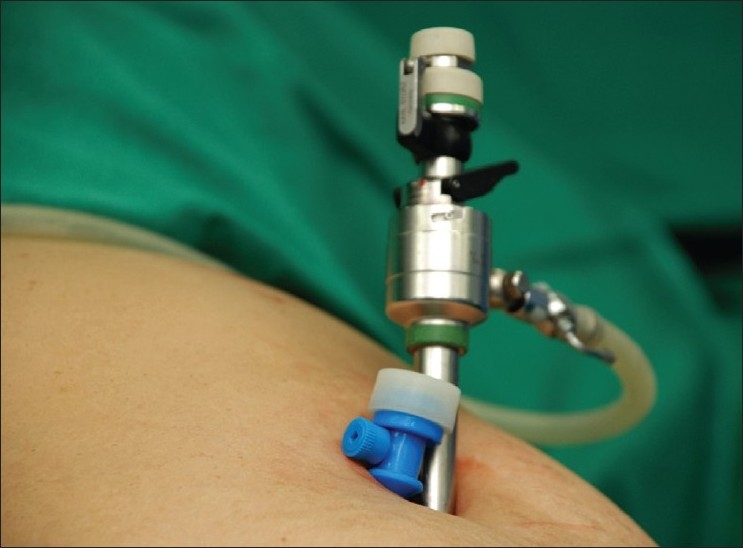
Two ports (10 and 5 mm) in the umbilical incision.

Our preference is for a transumbilical horizontal incision. The umbilicus is everted and held with two-toothed forceps in a cephalad and caudad position prior to making an incision of length 1.5-2 cm. This is deepened through the fat and the flaps are undermined to expose the fascia over a distance of 2-2.5 cm. The left edge of the skin incision is retracted and a fascial stab incision is made. A Verres needle is introduced through this incision and after confirmation of its intraperitoneal position, CO_2_ pneumoperitoneum is induced and maintained at 12 mm Hg. A 10-mm sharp-tipped port is placed in the peritoneal cavity after extending the fascial incision slightly. The preliminary step is a careful diagnostic laparoscopy. Particular attention is paid to the area around the umbilicus to exclude unsuspected omental or bowel adhesions. The right edge of the skin incision is then retracted, a stab incision made on the fascia and a low-profile 5-mm port is introduced [Figure 3]. Thus, the two ports are separated by a fascial bridge of about 5 mm, allowing the operating port to move without collision with the optic port. At this stage, the patient’s position is changed to an anti-Trendlenberg one with a left-sided tilt. Exaggerated tilts than those used in MLC may be required to obtain satisfactory exposure of the Calot’s triangle.

### Placement of traction sutures

This forms the key step of the SILC. Use of the “puppeteer” technique using traction sutures has been described previously.[2] At the beginning of the procedure, a grasper or a dissector is used to move the omentum away from the right upper quadrant so as to obtain a view of the fundus of the gallbladder. Flimsy omental adhesions, if present, may be teased off at this stage.

**Figure 4:**
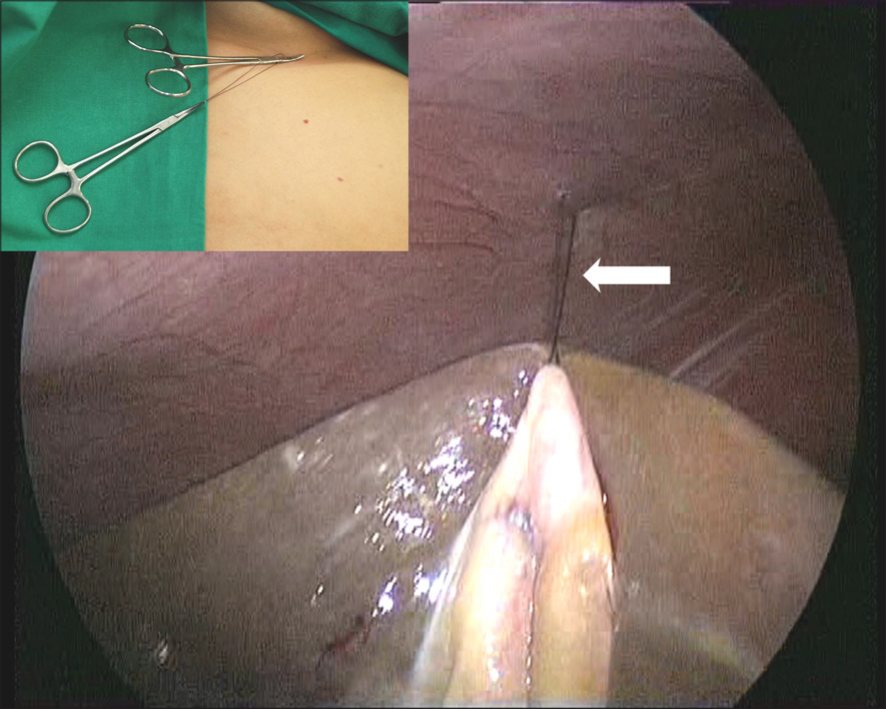
Cephalad elevation of the gallbladder via a fundal traction suture (arrow). Inset shows the traction suture held with hemostats.

We use a strand of 1-0 monofilament nylon on a 60-mm straight needle (Ethilon, Johnson and Johnson, Mumbai, India) for placing the traction sutures. The needle is introduced laterally through one of the intercostal spaces above the level of the costal margin. A laparoscopic needle holder brings the needle into the peritoneal cavity and places it on the omentum. The needle is then regrasped at its midpoint, a bite of the fundus of the gallbladder is taken and the needle is driven out through the same intercostal space. The needle is retrieved using an open needle holder, and the suture is pulled out leaving two ends of 5-6 cm. The suture is divided and a haemostat is applied to both ends close to the skin, resulting in elevation of the gallbladder fundus [Figure 4]. In our experience, if both passes of the sharp, straight needle through the intercostal space are at right angles to the chest wall, then pneumothorax does not occur. If the omental adhesions to the fundus are dense, they can be easily dissected at this stage once a strong counter-traction on the fundus is obtained.

**Figure 5:**
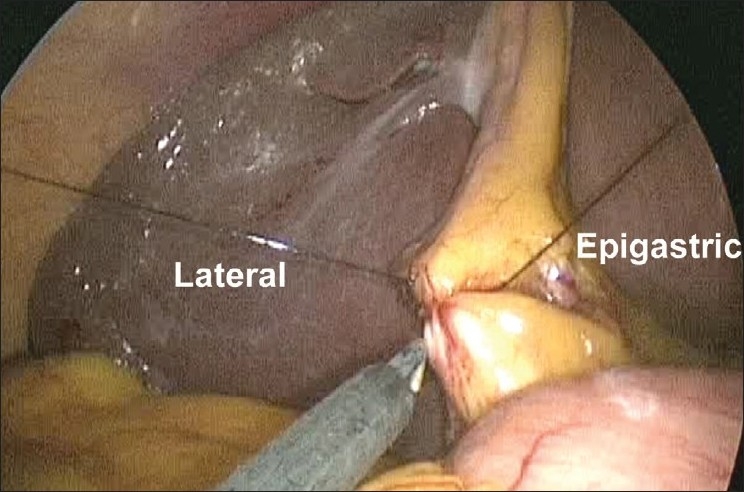
Suspension of the gallbladder with a loop suture on the Hartmann’s pouch. Epigastric = suture entering from the epigastrium. Lateral = suture exiting from lateral abdominal wall.

The second traction suture placed on the Hartmann’s pouch area as close to its junction with the cystic duct as possible. The straight needle is introduced high up in the epigastrium just to the right of the falciform ligament, grasped with the laparoscopic needle holder and driven through the Hartmann’s pouch. The needle is retrieved and a second pass is made to form a loop on the gallbladder. The needle and the suture are then passed through the loop and pulled. This locks the loop on the gallbladder. The needle exits the abdominal wall laterally [Figure 5]. Hemostats are placed on both ends of the suture. The gallbladder is now “suspended” on a length of suture, allowing its medial and lateral rotation in a manner identical to that during a multi-port cholecystectomy. The surgeon is thus able to carry out a two-handed dissection.

### Dissection of the Calot’s triangle

**Figure 6:**
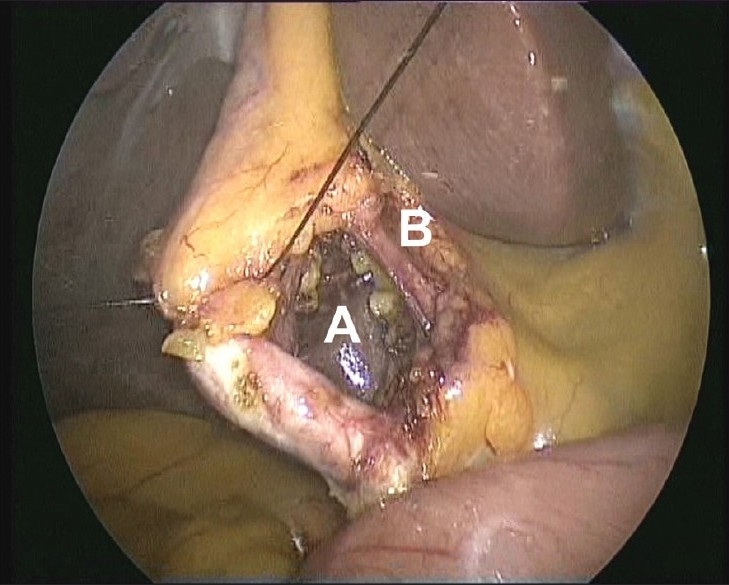
Critical view showing the window between the cystic duct and artery (A) and between the cystic artery and liver (B).

The posterior peritoneum is divided to free the Hartmann’s pouch. This is followed by further dissection of the anterior and posterior peritoneal leaves overlying the Calot’s triangle with the help of a hook and/or a Maryland dissector. The cystic artery and the cystic duct are skletonised - the endpoint of this dissection is obtaining a “critical view” [Figure 6]. Several aspects that add to the safety of this dissection merit highlighting. First, the insulation of operating instruments used in conjunction with diathermy must be scrupulously checked, as the trajectory of the instrument is such that the shaft may inadvertently come in contact with a loop of bowel outside the operative field. Second, the telescope must be withdrawn to observe the entry and exit of the operating instruments that travel parallel and close to bowel. Most importantly, the surgeon should not hesitate to “convert” the SILC to a LC by adding one or more 5-mm ports when faced with a gallbladder difficult to grasp, an unclear anatomy or a complication such as bleeding.

### Control of the cystic artery

**Figure 7:**
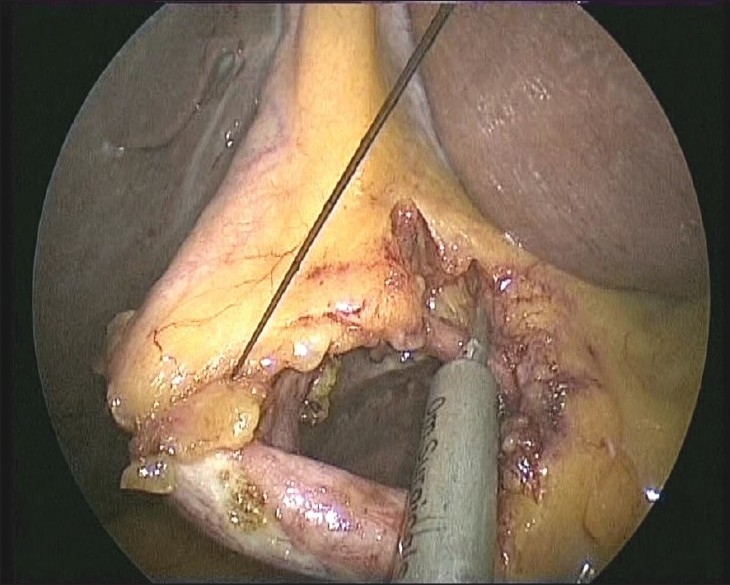
Cystic artery being clipped with a reusable 5-mm clip applicator.

The two windows in the Calot’s triangle are dissected more widely than during an LC so as to safely observe the tips of the instruments controlling the artery and the duct. We prefer to clip the cystic artery with a 5- or 10-mm reusable clip applicator [Figure 7]. When the latter is used, the telescope is temporarily shifted to the 5-mm port. Subsequently, the cystic artery is divided.

### Control of the cystic duct

**Figure 8:**
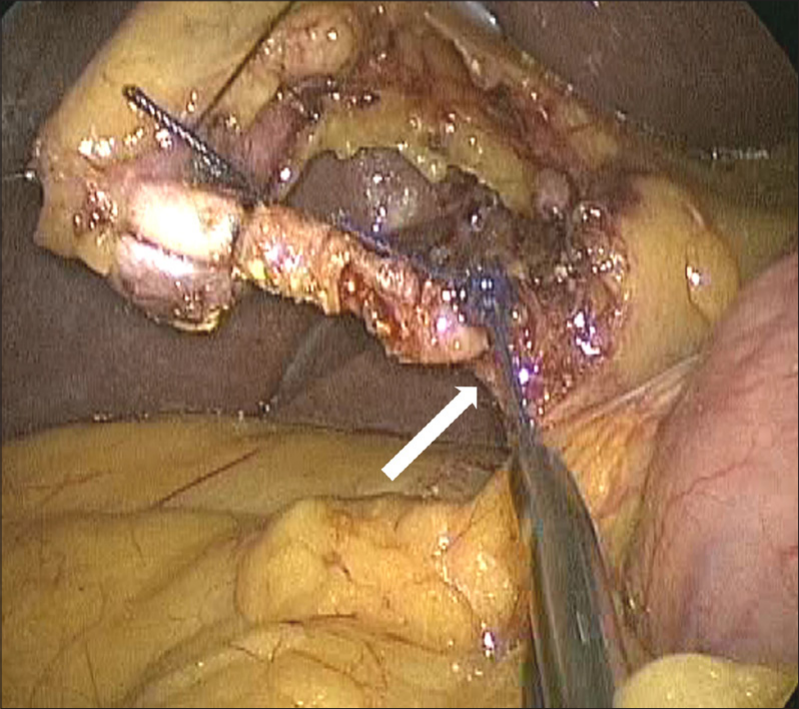
Cystic duct being ligated using an extracorporeal knot (arrow).

**Figure 9:**
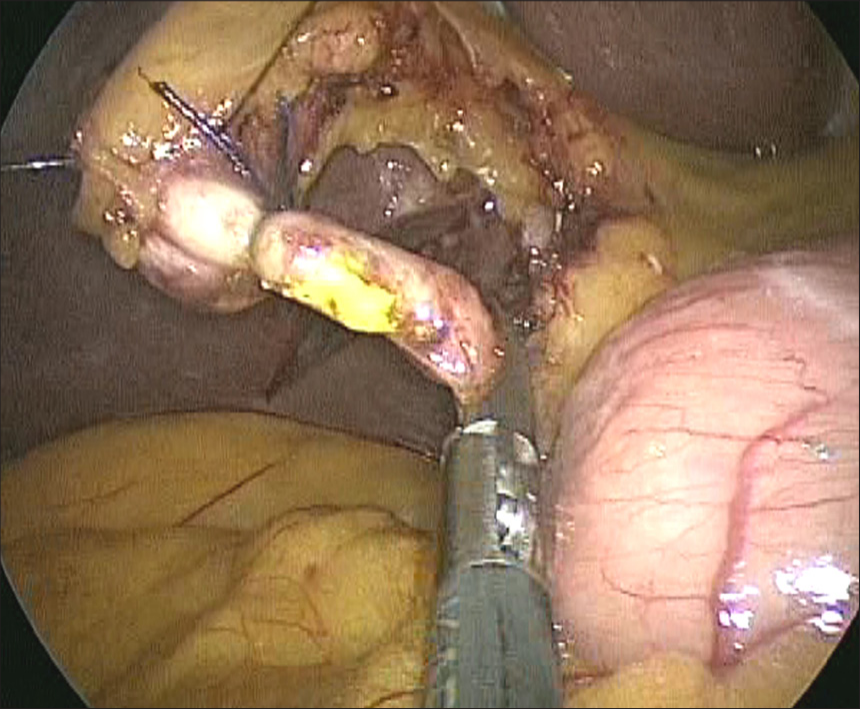
Stones milked out from a partly opened cystic duct.

If the cystic duct appears narrow, it is clipped thrice with 10-mm clips and divided. If the duct appears wide, we prefer to pass a No 1 polyglactin suture around it, exteriorise the same and fashion an extracorporeal Melzer’s knot. This knot is then snugged down onto the cystic duct with the help of a metal knot-pusher [Figure 8]. The duct is divided between two extracorporeally tied ligatures. If there is a suspicion of an impacted stone in the cystic duct, it is controlled on the gallbladder side, divided partially to allow the stone to be milked out [Figure 9], and then the stump is ligated using extracorporeal knotting. A 10-mm port is convenient for introducing a spoon forceps for the retrieval of stones from cystic duct or those that may spill from the gallbladder if it is perforated during dissection. The divided ends of the cystic artery and duct are carefully inspected to confirm their secure closure.

### Dissection of the gallbladder

Alternating medial and lateral rotation of the gallbladder using the ends of the suture placed on Hartmann’s pouch allows dissection of the gallbladder from the liver bed using a diathermy hook. Prior to the final detachment of the gallbladder, meticulous haemostasis in the liver bed is confirmed and the subhepatic space lavaged with saline. The fundal traction suture is loosened and the gallbladder is freed from the liver.

### Specimen extraction

**Figure 10:**
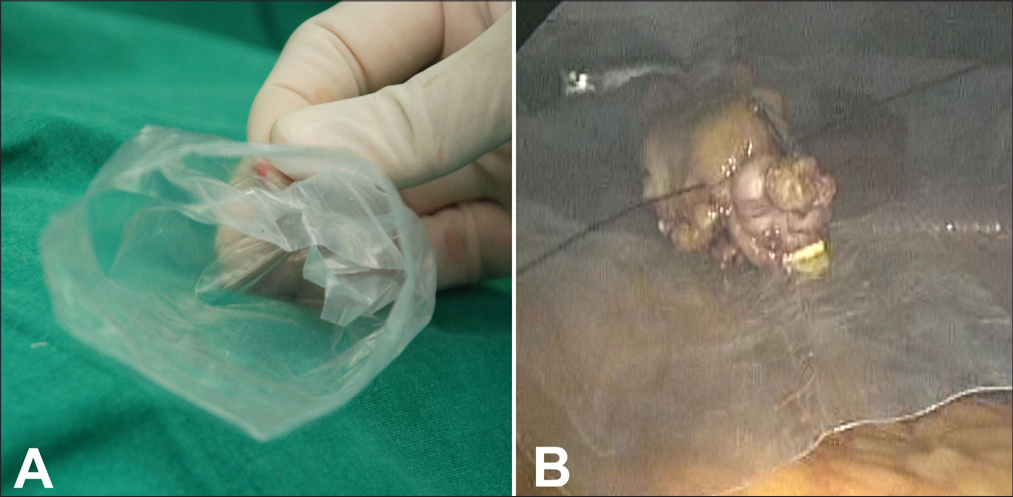
Everted plastic pouch stays open (A) and facilitates introduction of the gallbladder (B) for easy extraction.

We prefer to use a sterile, inexpensive plastic pouch that is turned inside out. The pouch is rolled up, introduced in the abdomen through the 10-mm port and opened on the superior surface of the liver. The mouth of the everted bag stays open and aids in the one-handed introduction of the specimen into it [Figure 10]. With the telescope in the 5-mm port, the pouch is grasped with a heavy grasper and pulled into the trocar. The pneumoperitoneum is deflated and mouth of the pouch is pulled out of the fascial incision. If necessary, the fascial incision may be extended or the two fascial incisions joined to facilitate extraction of the specimen.

### Closure of the incision

**Figure 11:**
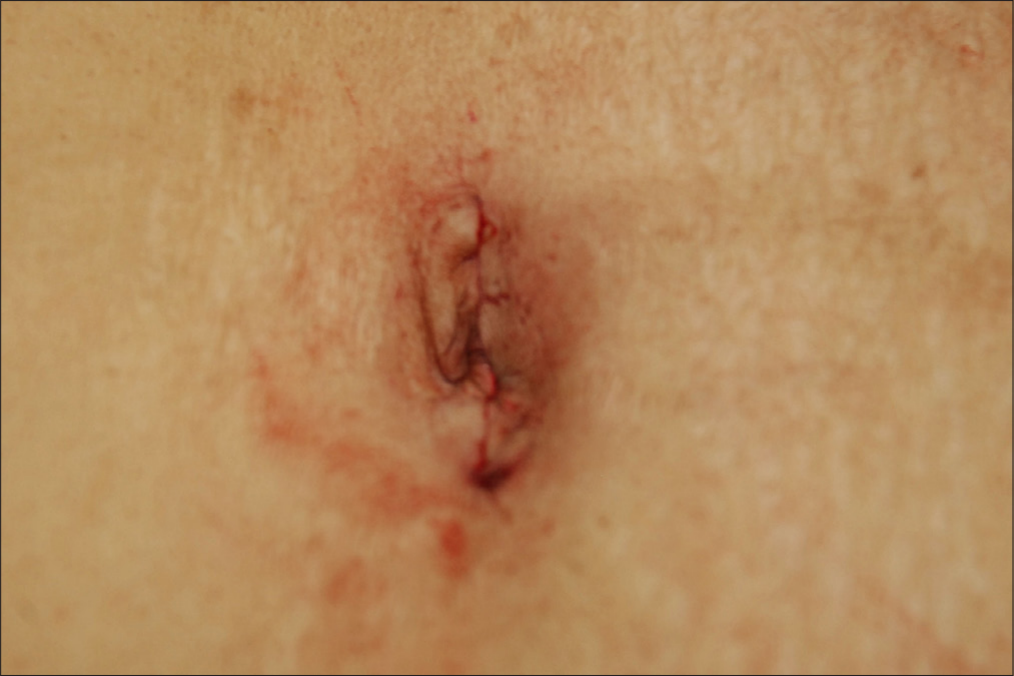
Final appearance of transumbilical incision closed with subcuticular suture.

Careful closure of the fascial incision is mandatory to prevent formation of port-site hernia. The edges of the fascial incision are identified, grasped and elevated using fine Kocher’s forceps. We prefer to close the fascia using two or more figure-of-eight sutures of a nonabsorbable suture material such as no 1 or 1-0 polypropylene. The fascia and the skin are infiltrated with a local anaesthetic and the skin is approximated with a running subcuticular suture [Figure 11].

### Postoperative course

The postoperative care is identical to that of patients undergoing a MLC. Intravenous analgesics and anti-emetics are administered for the duration of hospital stay. Patients are allowed to ambulate and take liquids within 6-8 hours of surgery and discharged within 24-48 hours. Oral analgesics are prescribed at discharge, but in our experience, most patients do not require analgesics beyond the second postoperative day.

## REVIEW OF THE LITERATURE

SILC is a relatively new technique and despite the enthusiasm of surgeons around the world for its application, data that can prove its superiority over the conventional multi-port cholecystectomy are scant. However, the literature related to SILC is evolving at a rapid pace. Recently, Antoniou *et al*. reported a systematic review of 29 studies, including a total of 1,166 patients undergoing SILC.[3] This review presents a number of salient features of this procedure.

### Patient demographics

Patients with a lower body mass index (BMI) were often considered as suitable candidates in most studies. Similarly, many studies included acute cholecystitis as exclusion criteria for offering SILC. This reflects the initial learning curve of SILC and is comparable to what was reported in the early literature pertaining to MLC.

### Surgical technique

A number of different techniques were described in terms of the number, type, and diameter of the trocars, the instruments and the method of gallbladder retraction and dissection of the Calot’s triangle. Many surgeons reported discomfort using roticulating instruments. Clashing of rigid instruments was not considered as a significant technical problem.

Undue reliance on technology, particularly on disposable ports and instruments, precludes the widespread application of the procedure. Our technique described here used standard instruments (barring the long laparoscope) for all cases of SILC. At the same time, the emphasis was on emulating the key “safety” steps of MLC, viz. adequate fundal and lateral traction, demonstration of the “critical view” and secure control of the cystic artery and cystic duct.

### Technical failure and morbidity

In the review by Antoniou *et al*.,[3] SILC was unsuccessful in 9.3% of the patients. The most common causes for failure were obscure anatomy at Calot’s triangle, inadequate exposure of the Calot’s triangle due to insufficient gallbladder retraction and inability to maintain pneumoperitoneum. Conversion to open surgery was required in 0.4% patients.

Intraoperative complication rates ranged from 0% to 20%, with a cumulative rate of 2.7%. The most common intraoperative complications were gallbladder perforation/bile spillage and haemorrhage, whereas the most common postoperative complications were haematoma, bile leakage, and residual choldocholithiasis.

Recently, Chiruvella *et al*. reported an instance of combined Bismuth type III bile duct and right hepatic artery injury in a patient undergoing SILC.[4] This case underscores the fact that surgeons undertaking SILC should receive adequate training in the procedure and, at all times, have a low threshold for conversion, i.e. for placement for additional port(s) (or indeed conversion to open surgery) to safely complete the procedure.

### Outcome analysis

The review indicated a lower rate of complication in studies enrolling patients with a mean age less than 45 years. The operative times were longer in studies enrolling patients with a BMI of >30 kg/m^2^. Inclusion of patients with acute cholecystitis did not increase the complication rates, but the operative times tended to be longer in studies that included patients with acute cholecystitis.

The authors of the review highlighted that although a meta-analysis of about 78,747 patients undergoing MLC showed the incidence of wound infection of 1.1% and wound haematoma rate of 0.6%,[5] the current review showed that wound complications occurred in 2.1% of the patients undergoing SILC. Concerns have also been raised about the likely higher incidence of port-site hernias due to the use of multiple closely placed fascial incisions through a narrow area. A “Swiss-cheese” configuration of 5-mm fascial defects and pressure necrosis of the tissues due to placement of tight-fitting access devices are factors to be considered. Careful and secure closure of fascial defect at the umbilicus is mandatory to prevent this complication. Moreover, a long-term follow-up is required to ensure that a higher incidence of port-site hernias does not mar the short-term benefits in terms of lower pain and cosmesis after SILC.

Only two randomised trials comparing SILC with MLC have been reported in the literature so far. Lee *et al*.[6] randomised 70 patients to SILC and MLC groups (35 in each group). SILC was performed using Quadraport Laparoscopic Access Device (LAGIS, Taichung County, Taiwan) and the MLC was carried out using a 10-mm umbilical port for the endocamera and three 3-mm ports for instruments. Surgical pain scores, analgesic requirements, and time to return to work were similar in both groups. There was a statistically significant difference in favour of patients undergoing SILC in terms of the hospital stay, shorter wound length, and better cosmetic appearance. The MLC procedures required shorter time to perform than the SILC operations. Authors concluded that although SILC is superior to MLC in terms of cosmesis, SILC are MLC were equal in terms of postoperative pain and analgesia. Tsimoyiannis *et al*. randomised 40 patients into two groups of 20 each who underwent SILC and MLC.[7] They observed significantly lower scores for abdominal pain in patients undergoing SILC after the first 12 hours and for shoulder pain after first 6 hours. Total pain after the first 24 hours was non-existent in the SILC group and the analgesic requirements were also significantly lower. As the number of patients included in this study is small, it is hard to conclusively confirm the superiority of SILC over MLC. A number of other trials comparing the two procedures are currently underway, and whether SILC is conclusively superior to MLC will become apparent once the results of these trials are published.

## OUR EXPERIENCE WITH SILC

One of the authors (DB) attempted SILC in 110 patients between August 2009 and October 2010 and completed it successfully in 105 (unpublished data). The procedures were performed for elective indications (N=84) as well as for acute cholecystitis (N=26). Conventional laparoscopic instruments as described above were used in all the patients. One additional 5-mm port had to be placed in four patients. This was required in two patients with acute cholecystitis to allow the Hartmann’s pouch to be grasped adequately for lateral traction. In two other patients a port was required to assist suturing of a) a duct of Luschka in the liver bed and b) stump of a sessile gallbladder joining the bile duct. One patient with acute cholecystitis required conversion to four-port cholecystectomy. Almost 30% patients who had elective SILC could be discharged the day after surgery and over 80% patients reported not requiring the oral analgesics they were prescribed at discharge. There were no postoperative or wound-related complications and all the patients were uniformly pleased with the cosmetic result.

### Summary

We have presented a technique of SILC using standard laparoscopic instruments that emulates all the steps of a safe multi-port cholecystectomy. We found that the technique has a relatively short learning curve and is reproducible. Preliminary studies show that SILC carries certain benefits over MLC. However, SILC should be considered a technique under evolution and further larger studies are required before it can be accepted as a replacement to MLC.
